# A Multi‐Scale Structural Engineering Strategy for High‐Performance MXene Hydrogel Supercapacitor Electrode

**DOI:** 10.1002/advs.202101664

**Published:** 2021-08-02

**Authors:** Xianwu Huang, Jiahui Huang, Dong Yang, Peiyi Wu

**Affiliations:** ^1^ State Key Laboratory of Molecular Engineering of Polymers and Department of Macromolecular Science Laboratory for Advanced Materials Fudan University Shanghai 200433 China; ^2^ State Key Laboratory for Modification of Chemical Fibers and Polymer Materials College of Chemistry Chemical Engineering and Biotechnology Center for Advanced Low‐Dimension Materials Donghua University Shanghai 201620 China

**Keywords:** 3D printing, energy density, hydrogel, multi‐scale, MXene, supercapacitor electrodes

## Abstract

MXenes as an emerging two‐dimensional (2D) material have attracted tremendous interest in electrochemical energy‐storage systems such as supercapacitors. Nevertheless, 2D MXene flakes intrinsically tend to lie flat on the substrate when self‐assembling as electrodes, leading to the highly tortuous ion pathways orthogonal to the current collector and hindering ion accessibility. Herein, a facile strategy toward multi‐scale structural engineering is proposed to fabricate high‐performance MXene hydrogel supercapacitor electrodes. By unidirectional freezing of the MXene slurry followed by a designed thawing process in the sulfuric acid electrolyte, the hydrogel electrode is endowed with a three‐dimensional (3D) open macrostructure impregnated with sufficient electrolyte and H^+^‐intercalated microstructure, which provide abundant active sites for ion storage. Meanwhile, the ordered channels bring through‐electrode ion and electron transportation pathways that facilitate electrolyte infiltration and mass exchange between electrolyte and electrode. Furthermore, this strategy can also be extended to the fabrication of a 3D‐printed all‐MXene micro‐supercapacitor (MSC), delivering an ultrahigh areal capacitance of 2.0 F cm^–2^ at 1.2 mA cm^–2^ and retaining 1.2 F cm^–2^ at 60 mA cm^–2^ together with record‐high energy density (0.1 mWh cm^–2^ at 0.38 mW cm^–2^).

## Graphical abstract

A strategy of multi‐scale structural engineering is proposed to fabricate high‐performance MXene hydrogel supercapacitor electrode with both vertically ordered macrostructure and the proton‐intercalated microstructure. This strategy also can be extended to the fabrication of 3D‐printed all‐MXene micro‐supercapacitor, delivering an ultrahigh areal capacitance together with record‐high energy density.

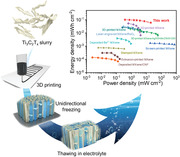

## 1 Introduction

The rapid development of electronic technology accompanied by the impendent shortage of petroleum fossil stocks and environmental pollution calls for advanced energy storage devices. In particular, electrochemical capacitors (ECs, also denoted as supercapacitors) that can store electrical energy harvested from intermittent sources and supply electrical energy rapidly have been extensively explored and achieved rapid development in recent years.^[^ ^1^, ^2^, ^3^ ^]^ However, considering the need to efficiently power ubiquitous portable electronics or even larger equipment in our day‐to‐day lives, it is necessary yet challenging to improve their energy density. Viewing from the charge storage mechanism and key components of ECs, this improvement requires not only excellent charge‐storage capability of the electrode materials, but also impactful strategies to optimize the design of the electrode structure.^[^ ^4^, ^5^ ^]^ Two‐dimensional (2D) nanomaterials have gradually become potential electrode materials in supercapacitor due to their built‐in electronic properties, large specific surface area, and abundant electrochemical active sites.^[^ ^6^, ^7^, ^8^, ^9^ ^]^ But these promising properties often come at a cost: different from the assembly of zero‐dimensional (0D) or one‐dimensional (1D) materials, 2D materials as electrodes tend to lie flat on the substrate and assemble into a compact‐stacking structure, resulting in highly tortuous ion pathways orthogonal to the current collector, which impede ion transport and cause sluggish kinetic.^[^ ^10^ ^]^ Therefore, two main strategies have been proposed to address the self‐restacking issue: 1) expanding the interlayer space by introducing the intercalated pre‐pillaring agent and 2) designing ordered or porous three‐dimendional (3D) structure using 2D flakes as the building blocks.

As a burgeoning member of the 2D material family, transitional metal carbide or nitride, known as MXene, shows compelling performance in electrochemical energy storage.^[^ ^11^, ^12^ ^]^ The standard notation of MXenes is M*_n_*_+1_X*_n_*T*_x_*, in which M represents the transition metals (Ti, Mo, Cr, Zr, V, etc.) and X represents the carbon or nitrogen. Nevertheless, similar to other 2D materials, aggregation and self‐restacking of flexible MXene flakes are usually inevitable during the electrode fabrication due to strong van der Waals interactions and hydrogen bonds between adjacent nanosheets. To address the problem, intercalated agents like CTAB,^[^ ^13^ ^]^ TAEA,^[^ ^14^ ^]^ Fe_2_O_3_,^[^ ^15^ ^]^ Be^2+^,^[^ ^16^ ^]^ and conducting polymers^[^ ^17^, ^18^, ^19^, ^20^ ^]^ have been introduced to expand the interlayer spacing of MXene flakes to counteract the effect of the self‐stacking propensity. The expanded interlayer spacing facilitates ion accessibility at the microscopic scale because MXene is a pseudocapacitive intercalation material in acidic electrolyte, in which oxygen‐containing functional groups undergo protonation with the intercalated H^+^, accompanied by a transformation in the titanium oxidation state.^[^ ^7^, ^21^ ^]^

On the other hand, at the macroscopic scale, sufficient electrolyte infiltration and the mass exchange between electrolyte and electrode are crucial for the rate performance and cycling stability, which calls for the ordered channels or porous structures that afford through‐electrode ion or electron transportation pathways.^[^ ^22^ ^]^ Various strategies, including template‐based method,^[^ ^23^, ^24^ ^]^ mechanical shear,^[^ ^25^ ^]^ lyophilization,^[^ ^26^, ^27^ ^]^ and gelation,^[^ ^28^, ^29^ ^]^ have been developed to regulate well‐defined structures of electrodes. For example, MXene flakes were assembled to liquid crystals with surfactants and then aligned vertically by applying shear stress.^[^ ^25^ ^]^ MXene hydrogel supercapacitor electrode can be obtained by fast gelation initiated by Fe^2+^.^[^ ^28^ ^]^ A strategy of in situ ice template was also performed to fabricate a 3D porous MXene/CNT aerogel electrode.^[^ ^23^ ^]^ In all three cases, a significant improvement in rate capability and cycle lifetime was observed.

Inspired by two aforesaid strategies, the design of electrode architectures over multiple length scales from microscopic to macroscopic is supposed to promote MXenes to reach exceptional performance. Herein, we develop an impactful strategy to fabricate a Ti_3_C_2_T*_x_* MXene hydrogel electrode based on multi‐scale structural engineering. After unidirectional freezing, MXene slurry can form a free‐standing and ordered hydrogel by thawing in the sulfuric acid electrolyte. This hydrogel can be directly used as the electrode without the lengthy freezing‐drying process. From microscopic to macroscopic dimensions, the synergistic effect of the intercalation of H^+^ and vertical alignment of MXene flakes enables sufficient ion diffusion and fast ion transport through the electrode, rendering ultrahigh capacitance performance (393 F g^−1^ at 5 mV s^–1^) and good rate performance (198 F g^−1^ at 1000 mV s^–1^). Furthermore, the strategy presented here is scalable and can be extended to 3D‐printing all‐MXene micro‐supercapacitors (MSCs), delivering an ultrahigh areal capacitance of 2.0 F cm^–2^ at 1.2 mA cm^–2^ and retaining 1.2 F cm^–2^ at 60 mA cm^–2^. A maximum energy density of 0.1 mWh cm^–2^ (0.38 mW cm^–2^) is achieved, higher than that of all ever‐reported MXene MSCs.

## 2 Results and Discussion

**Figure 1:**
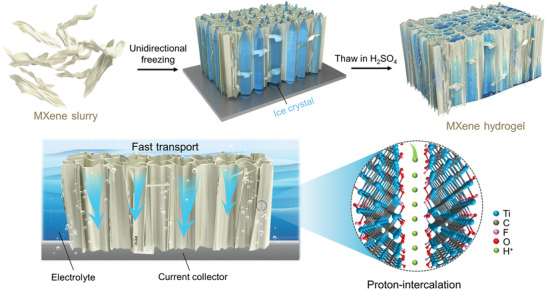
Schematic illustration of the multi‐scale structural engineering strategy (MSES) combining unidirectional freezing and thawing in H_2_SO_4_ to fabricate MXene hydrogel supercapacitor electrode.

**Figure 2:**
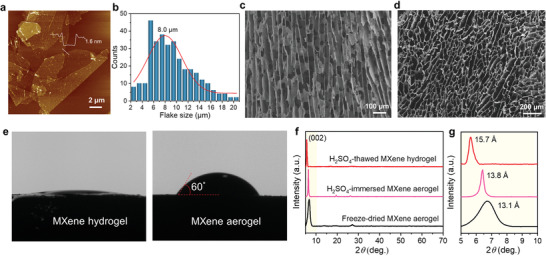
a) The AFM image of Ti_3_C_2_T*_x_* MXene flakes. b) The statistical flake size of MXene flakes according to TEM images. c) The sectional‐ and d) top‐view SEM images of ordered MXene hydrogel. e) Water contact angles of MXene hydrogel and aerogel. f) XRD patterns and g) amplified patterns of H_2_SO_4_‐thawed MXene hydrogel, H_2_SO_4_‐immersed aerogel, and freeze‐dried aerogel, respectively.

The multi‐scale structural engineering of the MXene hydrogel electrode is schematically represented in **Figure** **1**, including three steps: 1) preparing delaminated MXene slurry; 2) unidirectional freezing to induce pre‐assembly of MXene; 3) thawing in sulfuric acid electrolyte. First of all, to ensure the integrity of the assembled electrode, large and high‐quality MXene flakes were prepared by freeze‐and‐thaw‐assisted (FAT) approach according to our previous research,^[^ ^30^ ^]^ with an average statistical lateral size of ≈8.0 µm and thickness of 1.6 nm as shown in AFM, TEM, and SEM (**Figure** **2**a,b; Figure S1, Supporting Information). Also, the obvious crystal lattices were observed via HRTEM and its corresponding selected area diffraction pattern (SAED) (Figure S2, Supporting Information) manifested the hexagonal crystal structure, indicating that the delaminated MXene flakes maintained the crystal integrity.^[^ ^30^ ^]^

To date, the unidirectional freeze‐casting method has been extensively applied for assembling MXene flakes into tailored aligned porous macrostructure in the field of electromagnetic interference (EMI),^[^ ^31^, ^32^ ^]^ solar desalination,^[^ ^33^ ^]^ and thermal management.^[^ ^34^ ^]^ Freeze casting generally involves the controlled solidification of a suspension, followed by the sublimation of water under reduced pressure. Optimally, in this work, the tedious lyophilization process can be ignored and replaced by directly thawing in sulfuric acid electrolyte, in which the structural framework is retained thanks to the optimized mass transfer and heat transfer. After unidirectional freezing, ice crystals growing up along the direction of the temperature gradient make MXene flakes align vertically and form ordered channels in the macroscopic structure (Figure 2c,d). During the well‐designed thawing process in 3 m H_2_SO_4_, on the one hand, the replacement of Li^+^ with protons effectively prevents the disassembly of Ti_3_C_2_T*_x_* assemblies by reducing the tendency of H_2_O intercalation and the hydration of Ti_3_C_2_T*_x_* nanosheets;^[^ ^35^ ^]^ on the other hand, the freezing point of the thawing solution is below −20 °C, at which the structure of ice is not affected,^[^ ^36^ ^]^ thus contributing to a free‐standing MXene hydrogel with vertically aligned macrostructure and proton‐intercalated microstructure. The massive protons enter the interlayer to counteract the van der Waals interactions and meanwhile participate in the pseudocapacitive electrochemical reaction,^[^ ^21^ ^]^ which prevent the restacking tendency of MXene flakes and lay the core foundation of realizing high‐performance supercapacitors. For comparison, unordered MXene hydrogel is obtained by the same method except for direct freezing in a fridge (−20 °C). Ordered MXene aerogel is prepared by unidirectional freezing followed by lyophilization. Admittedly, a good wetting property of electrolyte for electrode has a primary effect in rendering mass exchange more accessible. In contrast to ordered aerogel, the smaller water contact angle of MXene hydrogel demonstrates the faster infiltration of electrolyte (Figure 2e). Besides, X‐ray diffraction (XRD) patterns of MXene aerogel, H_2_SO_4_‐immersed MXene aerogel, and H_2_SO_4_‐thawed MXene hydrogel were collected. The (002) diffraction peaks for the three samples were located at 6.72^o^, 6.42^o^, and 5.6^o^, respectively. The corresponding interlayer spacings were 13.1, 13.8, and 15.7 Å according to the Bragg equation. That is, compared to MXene aerogel, our MXene hydrogel electrode has both aligned MXene flakes and enlarged interlayer spacings, affording unimpeded channels for enhanced ion accessibility and easing the tortuosity of the ion transport pathways to ameliorate the transport of charge storing ions through the electrode.

Notably, the ordered skeleton using MXene flakes as building bricks also functions as an electrical highway and a mechanical backbone. Under uniaxial compression, the long‐range orientation of the MXene bricks enhances the mechanical performance in the vertical direction, exhibiting a higher compressive modulus (110 kPa) than unordered hydrogel (14.6 kPa, Figure S3, Supporting Information) The mechanically stable electrode would lay the groundwork for cycling and rate performance.

**Figure 3:**
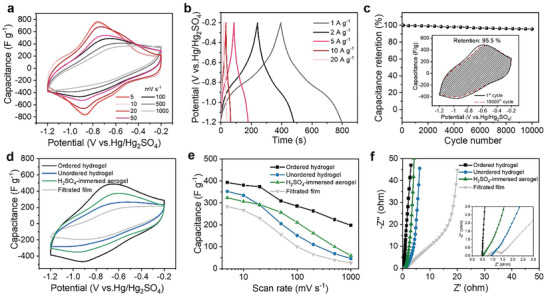
a) CV curves and b) GCD profiles of ordered MXene hydrogel at different scan rates and current densities, respectively. c) Cycling performance of ordered MXene hydrogel. Inset: comparison of the CV curves before and after 10 000 cycles (100 mV s^–1^). Comparison of the d) CV curves (100 mV s^–1^), e) rate performance, and f) EIS of ordered hydrogel, unordered hydrogel, H_2_SO_4_‐immersed aerogel, and filtrated MXene film, respectively.

The free‐standing ordered MXene hydrogel could be used directly as working electrodes without drying or any additives, and shows excellent wettability for the acidic electrolyte (3 m sulfuric acid). Its electrochemical performance was first measured in a Swagelok‐type three‐electrode setup with glassy carbon current collectors (Figure S4, Supporting Information). The cyclic voltammetry (CV) profiles at scan rates ranging from 5 to 1000 mV s^–1^ were recorded (**Figure** **3**a). A pair of redox peaks were observed at low scan rates and became broader at high scan rate, indicating the intercalated pseudocapacitive energy storage mechanism of Ti_3_C_2_T*_x_* in acidic electrolyte. The specific electrochemical reaction can be expressed as:^[^ ^37^ ^]^

(1) $${\mathrm{Ti}}_{3}C_{2}O_{x}{\left(\mathrm{OH}\right)}_{y}F_{z}+\delta e^{-}+\delta H^{+}\to {\mathrm{Ti}}_{3}C_{2}O_{x-\delta }{\left(\mathrm{OH}\right)}_{y+\delta }F_{z}$$

In addition, the galvanostatic charge–discharge (GCD) curves of MXene hydrogel at different current densities between −1.2 and 0.2 V (vs Hg/Hg_2_SO_4_) are shown in Figure 3b. The symmetric GCD curves deviate from the ideal isosceles‐triangle nature, which is one signature of MXene that store charge not only via charge double layer (EDLC) but also through pseudocapacitive mechanism on behalf of redox active surface.^[^ ^38^ ^]^ The cycling performance of the MXene hydrogel was tested at a scan rate of 100 mV s^–1^ as shown in Figure 3c. The highly overlapping CV curves (Figure 3c inset) shows the capacitance retention of 95.5% after 10 000 cycles, suggesting good cycling stability of MXene hydrogel.

To demonstrate the superiority of the ordered hydrogel electrode, unordered MXene hydrogel, MXene filtrated film, and ordered MXene aerogel were used as references. At the same scan rate (100 mV s^–1^), the ordered MXene hydrogel shows an obviously larger CV integration area than the other three reference samples, as shown in Figure 3d. To evaluate the rate performance, the gravimetric capacitances at different scan rates were calculated from the CV curves (Figure 3e; Figure S5, Supporting Information). Among these electrodes, the ordered MXene hydrogel electrode shows the highest gravimetric capacitance at various scan rates and exhibits the specific capacitance of 393.0 F g^–1^ at a scan rate of 5 mV s^–1^, markedly higher than those of unordered hydrogel (352. 2 F g^–1^), H_2_SO_4_‐immersed aerogel (323.5 F g^–1^), and filtrated film (283 F g^–1^). It is worth noting that the ordered MXene hydrogel electrode could still retain a high gravimetric capacitance of 198 F g^–1^ with an excellent rate capability (50.3% at 1000 mV s^–1^), much higher than those of unordered hydrogel (13.0%), H_2_SO_4_‐immersed aerogel (18.5%), and filtrated film (9%), emphasizing the importance of the synergy between the vertically aligned macrostructure and proton‐intercalated microstructure to expedite charge and ion transport. In contrast, the performance of MXene film is underperformed. This is because the serious self‐restacking propensity of MXene layers could increase the tortuosity and lose the unobstructed channels, thereupon severely impeding the accessibility of electrolyte ions and leading to an inadequate Faraday reaction.^[^ ^5^ ^]^ As shown in Figure 3f, the electrochemical impedance spectra (EIS) with a frequency range from 10^–2^ to 10^5^ Hz were performed to give further insights into the charge transfer and ion transport in the electrodes. Due to the difference in conductivity of these four samples, the series resistances (*R*_Ω_, real axis intercept) of the ordered MXene hydrogel and aerogel electrode with aligned architecture are slightly lower than those of MXene film and unordered MXene hydrogel. The semicircles in plots of hydrogel and aerogel become inappreciable in the high‐frequency region, manifesting that the interfacial charge‐transfer resistance (*R*_ct_) can be decreased greatly by introducing the 3D open architectures. In addition, the Nyquist plots of the filtrated film electrodes show a clear 45° Warburg‐type impedance element in the mid‐frequency region. Contrastingly, plots of the ordered MXene hydrogel electrode are nearly vertical at all frequencies, revealing the fast ion diffusion and ideal capacitive behavior. The results of EIS are highly consistent with the rate performance. Thus, the rate capability of MXene hydrogel can be attributed to the vertically aligned architecture and increased interlayer spacing caused by the intercalation of protons between MXene layers. These characteristics synergistically create better kinetic performance by improving the diffusion and transportation of electrolyte ions and increase the utilizable surface area accessible to the ions.^[^ ^39^ ^]^

The charge storage mechanism can be further studied by analyzing the relationship between the peak current (*i*) and the scan rate (*v*) according to the power law *i* = *av^b^*,^[^ ^23^, ^40^ ^]^ in which *a* and *b* are parameters. The *b* value of 0.5 indicates a diffusion‐controlled process where capacitance is derived from battery‐type Faradaic intercalation, while the *b* value of 1 means the nondiffusion‐controlled capacitive process including EDLC capacitance and pseudocapacitance. As shown in Figure S6, Supporting Information, the *b* value of the MXene film and unordered MXene hydrogel electrode obviously deviate from 1 when the scan rate is higher than 50 mV s^–1^. In comparison, the *b* value of the ordered MXene hydrogel and ordered aerogel electrode remains close to 1 even at higher scan rates, meanwhile the *b* value of the ordered MXene hydrogel is apparently higher than that of ordered aerogel, indicating the dominant contribution from the nondiffusion controlled capacitive process associated with high ion accessibility induced by the 3D architecture and proton‐intercalated micro‐structure.

**Figure 4:**
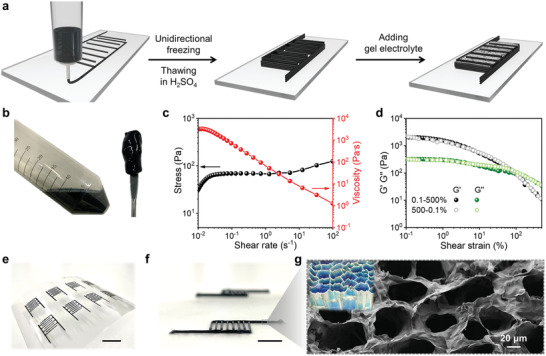
a) The fabrication process of 3D‐printing all‐MXene MSC via MSES. b) The digital photographs of MXene slurry. c) Shear‐thinning behavior of the MXene inks (stress and viscosity against different shear rates). d) The oscillatory measurements (1 Hz) of the MXene ink, which sweeps from 0.1% to 500% and back to 0.1% strain. e,f) Photographs of 3D‐printed MXene MSC, scale bar is 1 cm. g) The top‐view SEM image of MXene hydrogel MSC.

Furthermore, thanks to the weak gelation property of MXene slurry,^[^ ^41^ ^]^ it is possible to create oriented microstructures even at high concentrations, this strategy presented here is scalable and can be extended to the additive manufacturing of MXene micro‐supercapacitors (MSCs) as shown in **Figure** **4**a. As key characteristics for extrusion‐based direct ink writing (DIW) printing, certain rheological property of the printing ink is required to ensure smooth extrusion of the filament with good shape fidelity. At a high concentration (50 mg mL^−1^), the MXene slurry forms a viscous ink with a viscosity of 3.3 × 10^3^ Pa s and without aggregates (Figure 4b). The viscosity of MXene ink decreases as the shear rate increases, displaying a typical shear‐thinning thixotropic behavior (Figure 4c). Figure 4d displays the storage modulus (*G*′) and loss modulus (*G*″) of MXene ink as a function of shear strain, at a relative low shear strain ranging from 0.1% to 50%, plateaus of *G*′ are an order of magnitude higher than *G*″, which shows predominantly solid‐like behavior. After the intersection of *G*′ and *G*″ at around 100% shear strain, that is yield point, the moduli reversibly decrease, which means that the highly percolated MXene network breaks and exhibits liquid‐like properties (*G*″ > *G*′). This feature allows continuous extrusion through micron‐sized nozzles at a relatively low pressure. Once squeezed, it can recover the solid‐like behavior with high shape retention. The good recoverability of MXene ink is evaluated by strain sweep measurements from 0.1% to 500% and back to 0.1% strain, with the measured curves almost overlapping (Figure 4d). Therefore, the shear‐thinning behavior during extrusion and shape fidelity after extrusion are well appropriate for 3D printing free‐standing architectures in a large scale (Figure 4e,f; Movie S1, Supporting Information). Unidirectional freezing (Figure S7, Supporting Information) and thawing in H_2_SO_4_ were subsequently applied to assemble MXene flakes into a vertically‐oriented 3D scaffold infiltrated with acidic electrolyte. Finally, H_2_SO_4_‐PVA gel electrolyte was dropped on the interdigital patterns of electrode to ensure an adequate supply of electrolyte and complete the fabrication of solid‐state MSCs. The optical microscopy and scanning electron microscopy (SEM) were performed to investigate the morphologies and microstructures of the 3D printed and vertically aligned interdigitated electrodes. Optical microscopic images show the even distribution of interdigital finger gaps (≈610 µm) and printed filaments (≈350 µm in diameter, Figure S8, Supporting Information). As shown in Figure 4g, the top‐view SEM image of the MSC confirms the honeycomb‐like compartmental architecture due to the oriented growth of ice crystals along the thickness direction during the unidirectional freezing.

**Figure 5:**
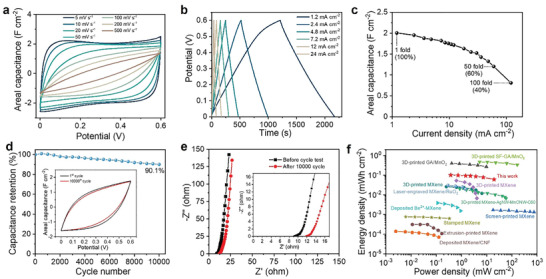
a) CV curves and b) GCD profiles of 3D‐printing MXene MSC at different scan rates and current densities, respectively. c) Rate performance of MXene hydrogel MSC. d) Cycling performance of MXene hydrogel MSC. Inset: comparison of the CV curves before and after 10 000 cycles (100 mV s^–1^). e) The EIS of MSC before and after cycle test. f) Ragone plot of the 3D‐printed MXene MSC via MSES comparing with other reported values.

In order to demonstrate the potential of MSES in fabricating MSC, the electrochemical performance of the 3D‐printed MSC was investigated by a two‐electrode configuration. **Figure** **5**a shows the CV curves of the MXene hydrogel MSC device at different scan rates, ranging from 5 to 500 mV s^–1^. The assembled demonstrator shows a quasi‐rectangular CV curve shape (5–200 mV s^–1^) without distinct peaks, revealing its ideal capacitive behavior. GCD curves for the MXene hydrogel MSC at different current densities are shown in Figure 5b. The triangular‐curve charging‐discharging profile confirms typical capacitive characteristics observed by CV curves and reveals the efficient charge storage ability. The areal capacitance, as a reliable benchmark, can assess the performance of MSCs. The maximum areal capacitance of MSC derived from CV curves is 1.98 F cm^–2^ at 5 mV s^–1^ (Figure 5a). In Figure 5c, the values achieved by GCD curves are comparable and can reach as high as 2.0 F cm^–2^ at 1.2 mA cm^–2^ and maintain over 60% (1.2 F cm^–2^) when the current density increases to 50‐fold (at 60 mA cm^–2^). Further, the retention of areal capacitance is about 40% when elevating the current density by 100 times (120 mA cm^–2^). The high areal capacitance and predominant rate performance overmatch state‐of‐the‐art MXene‐based MSCs fabricated by other methods as shown in Table S1, Supporting Information.^[^ ^16^, ^42^, ^43^, ^44^, ^45^, ^46^, ^47^, ^48^ ^]^ Moreover, in order to demonstrate the durability, MXene hydrogel MSC was cycled at 100 mV s^–1^ for 10 000 cycles. The capacitance retains at 90% compared to the initial capacitance (Figure 5d), showing excellent cycling performance. As shown in Figure 5e, EIS was applied to further elucidate the kinetics of electron and ion transportation of MSC. The Nyquist plot shows an ESR of 8.9 Ω based on the intercept of the curve with the real axis at high frequency region. After repeated CV tests, the resistance increases to 11.8 Ω because of the increase in the resistance of the electrolyte (inset of Figure 5e). However, no obvious semicircle is observed, suggesting that the *R*_ct_ can be neglected. Notably, the Nyquist plots both exhibit near vertical curves in the low frequency range, indicative of ideal capacitive behaviors and fast ionic diffusion, which agrees well with the CV and GCD results. This excellent ion transport behavior can be largely attributed to the ordered network structure in the 3D‐printed electrodes, which provides unimpeded pathways and improves electrolyte‐electrode contact, resulting in high ionic conductivity and low resistance to diffusion. Figure 5f enumerates the Ragone plots of recent works related to MSCs fabrication based on MXene and other materials. The 3D‐printed MXene MSCs fabricated via our strategy possesses a maximum energy density of 0.1 mWh cm^–2^ (0.38 mW cm^–2^), and the energy density is maintained at 0.06 mWh cm^–2^ at a high power density of 19.7 mW cm^–2^, which are orders of magnitude higher than that of all ever‐reported MXene MSCs fabricated by 3D‐printing, screen‐printing, stamping, and so on. It is worth noting that the performance of MXene hydrogel MSC can be further improved by optimizing the finger distance, finger numbers, and active materials load.^[^ ^49^ ^]^ Besides, the narrow voltage window (0.6 V) imposes restriction on the energy density when applying an aqueous electrolyte. In Ragone plots, there is still a considerable room for improvement for MXene related MSC in contrast to the 3D‐printed graphene SC.^[^ ^50^, ^51^ ^]^ Therefore, the use of nonaqueous electrolyte^[^ ^52^ ^]^ and asymmetric‐printing configurations,^[^ ^44^ ^]^ combining with our strategy, could unite in boosting the energy density.

## 3 Conclusions

In summary, a strategy adopting multi‐scale structural engineering to prepare MXene hydrogel supercapacitor electrode was proposed. Unidirectional freezing enables large MXene flakes to assemble into a hierarchical architecture with vertically aligned channels. By subsequently thawing in H_2_SO_4_, a freestanding MXene hydrogel electrode with proton‐intercalated microstructure can be obtained without further drying, while the ordered architecture can remain intact. Compared to directly freezing‐dried MXene aerogel and compact‐stacked MXene film, the as‐fabricated ordered MXene hydrogel is considered to be the optimal electrode, which possesses high capacitance (393 F g^–1^ at 5 mV s^–1^), and excellent rate capability (198 F g^–1^ at 1000 mV s^–1^). These improvements benefit from the vertically aligned MXenes architecture and increased interlayer spacing caused by the intercalation of protons between MXene layers, synergistically improving the diffusion and transport of electrolyte ions and increasing the surface area available for ions. Furthermore, when extending this method to the fabrication of 3D‐printed MXene MSC, the electrode also represents honeycomb‐like compartmental architecture, delivering an ultrahigh areal capacitance of 2.0 F cm^–2^ at 1.2 mA cm^–2^ and retaining 1.2 F cm^–2^ at 60 mA cm^–2^ together with record‐high energy density (0.1 mWh cm^–2^ at 0.38 mW cm^–2^). With integrated electrode architecture design over multiple length scales from microscopic to macroscopic, this strategy has demonstrated its merits of high energy density in energy‐storage applications and could innovate the conventional layer‐by‐layer stacking fabrication process of traditional supercapacitors.

## 4 Experimental Section

### Materials

Hydrofluoric (HF), hydrochloric (HCl), and sulfuric acid (H_2_SO_4_) were purchased from Shanghai Chemical Corp. Ti_3_AlC_2_ (MAX) powder with the average particle size of 200 meshes was provided by Jilin 11 Technology Co., Ltd. Polyvinyl Alcohol (PVA, *M*_w_ = 115 000) was purchased from Aladdin Co,. Ltd.

### Preparation of Delaminated Ti_3_C_2_T*_x_* MXene Slurry

MXene nanosheets were prepared according to the previously‐reported freeze‐and‐thaw (FAT) method.^[^ ^30^ ^]^ After four cycle times of FAT, the sediment composed of unreacted MAX and multilayer MXene was discarded by centrifuging at 1500 r.p.m. for 30 min and the suspension containing the delaminated MXene was collected by centrifuging at 3500 r.p.m. for 30 min. Then, the MXene slurry was obtained by the centrifugation of delaminated MXene dispersion at 9000 r.p.m. for 60 min. The mass ratio of MXene slurry was calculated by measuring the final mass of the known mass of slurry on a glass slide after vacuum drying. The concentration is about 50 mg mL^−1^. For comparison, MXene film was prepared by the filtration of delaminated MXene dispersion through a cellulose acetate membrane with a pore size of 3 µm, followed by vacuum drying.

### Preparation of Ordered MXene Hydrogel Electrode and Samples for Comparison

First, proper mass of MXene slurry (≈20 mg mL^−1^) was poured into a Teflon mold attached to a copper cylinder (cold finger), and the copper cylinder was immersed in the liquid nitrogen. Then the completely frozen MXene was rapidly taken out and thawed in 3 m H_2_SO_4_ with slight stir for 12 h to prepare ordered MXene hydrogel. For comparison, unordered MXene hydrogel was obtained in the same procedure besides freezing in the fridge (≈−20 °C). The ordered aerogel was prepared by unidirectionally freezing MXene slurry followed by freeze‐drying.

### Preparation of PVA/H_2_SO_4_ Gel Electrolyte

Briefly, 3 g PVA powder was added to 30 mL deionized water under vigorous stirring at 90 °C until the solution became transparent. After cooling down the as‐prepared PVA solution, 3 g of 98% H_2_SO_4_ was dropwise added to the solution under stirring at room temperature.

### 3D Printing and Assembly of All‐MXene MSCs

Extrusion‐based 3D printing of MXene ink was performed using a 3D Bio‐Architect working station (Regenovo). A computer program converted the input 3D model (drew by AutoCAD) into the machine command and controlled the extrusion of the ink with applied pressure of 0.12 MPa, and printing speed of 3–6 mm s^−1^. The diameter of the chosen conical needle tip was 210 µm. MSCs with interdigital patterns were obtained by layer‐by‐layer printing on PET substrate. After that, the 3D‐printed MSC was put on a PTFE plate with a thickness of a few centimeters and transferred onto the surface of a copper cylinder that was immersed in the liquid nitrogen. The active material loading of this MSC was about 5.2 mg cm^−2^. The completely frozen MSC was then immersed in 3 m H_2_SO_4_ for 12 h for thawing, H^+^ replacement, and electrolyte infiltration. After the formation of free‐standing 3D‐printed MSC, the redundant H_2_SO_4_ on the surface of PET was wiped away. Two silver wires were connected separately with two electrodes by conductive silver glue and covered by nail polish. After drying completely, the electrolyte gel was drop‐casted onto the interdigital electrode area.

### General Characterization Techniques

The morphology and structure of MXene nanosheets were observed by TEM (JEOL JEM2011 F Microscope) operated at 200 kV and AFM (Bruker Multimode 8). Scanning electron microscopy (SEM, Zeiss Gemini SEM500 FESEM) with an energy dispersive X‐ray detector was utilized to observe the morphology of aligned‐hydrogel. The crystalline structures of MXene film, aerogel, and hydrogel were examined by X‐ray powder diffraction (XRD, X'pert PRO PANalytical) with Ni‐filtered Cu K*α* radiation (40 kV, 40 mA). Optical microscope photograph was taken from the microscope (Leica, DM2500P). The water contact angle tests were performed on an optical contact angle meter (Kino, SL200KS). Compression tests were performed on a universal mechanical test machine at the deformation rate of 5 mm min^−1^ (Instron 5966, USA). The elastic modulus (*E*) was calculated by the average slope over 0–10% of strain from the typical stress (*σ*)–strain (*ε*) curve by the following formula:$\;E\;=\frac{\sigma }{\varepsilon }\;$. The rheological property of the MXene slurry for 3D printing was evaluated by an HAAKE MARS modular advanced rheometer with 25 mm parallel plate geometry at 25 °C. Viscometry measurements were conducted with shear rates ranging from 0.01 to 100 s^–1^. Continuous step strain tests were performed from 0.1% to 500% and back to 0.1% strain at the fixed frequency of 1 Hz.

### Electrochemical Characterization

Three‐electrode electrochemical measurements were performed in the Swagelok‐type cell by CHI 660E electrochemical workstation using glassy carbon as the current collector for both the working and counter electrodes. The aligned MXene hydrogel could be directly used as the working electrode and the over‐capacitive activated carbon films (YP50, Kuraray) were used as the counter electrodes. An aqueous mercury sulfate (Hg/Hg_2_SO_4_) electrode in saturated potassium sulfate (K_2_SO_4_) was used as the reference electrode. The PP membranes (Celgard 3501) was utilized as the separator and the electrolyte was 3 m H_2_SO_4_. Two‐electrode configuration was applied to test the 3D‐printing MSC. The operation potential is 1.0 V (−1.2 to −0.2 V vs Hg/Hg_2_SO_4_) and 0.6 V (0–0.6 V) for three‐ and two‐electrode system, respectively. Electrochemical impedance spectroscopy was recorded in the range from 10 mHz to 100 kHz with an amplitude of 5 mV. The calculation formulas for specific capacitance, areal capacitance, energy density, and power density are presented in the Supporting information.

## Conflict of Interest

The authors declare no conflict of interest.

## Supporting information

Supporting Information

Supplemental Movie 1

## Data Availability

The data that support the findings of this study are available from the corresponding author upon reasonable request.
