# Supplementary material for: A Multi‐Scale Structural Engineering Strategy for High‐Performance MXene Hydrogel Supercapacitor Electrode
Source: Adv Sci (Weinh). 2021 Aug 2;8(18):2101664. doi: 10.1002/advs.202101664 (PMC8456213; doi:10.1002/advs.202101664)
Supplement: Supplementary file 1 — Supporting Information [file ADVS-8-2101664-s001.pdf]

© 2021 Wiley-VCH GmbH

ADVANCED SCIENCE
Open Access

Supporting Information

for *Adv. Sci.*, DOI: 10.1002/advs.202101664

A Multi-Scale Structural Engineering Strategy for High-Performance MXene Hydrogel Supercapacitor Electrode

Xianwu Huang, Jiahui Huang, Dong Yang and Peiyi Wu**

Supplementary Materials for

A Multi-Scale Structural Engineering Strategy for High-Performance MXene Hydrogel Supercapacitor Electrode

Xianwu Huang, Jiahui Huang, Dong Yang and Peiyi Wu**

X. Huang, J. Huang, Prof. D. Yang, and Prof. P. Wu
State Key Laboratory of Molecular Engineering of Polymers Department of Macromolecular Science and Laboratory for Advanced Materials
Fudan University
Shanghai 200433, China

Prof. P. Wu
State Key Laboratory for Modification of Chemical Fibers and Polymer Materials College of Chemistry
Chemical Engineering and Biotechnology Center for Advanced Low-Dimension Materials
Donghua University
Shanghai 201620, China

Prof. D. Yang
E-mail: yangdong@fudan.edu.cn
Prof. P. Wu
E-mail: peiyiwu@fudan.edu.cn

Calculation formula of Specific capacitance ($C_{specific}$)Areal capacitance (C_{areal}), Energy density (E), and Power density (P)

$C_{specific} = (\int IdV)/(s\Delta Vm)$ (F g^{-1}) (3-electrode configuration); (1)

$C_{areal,CV} = (\int IdV)/(s\Delta VA)$ (F cm^{-2}) (2-electrode configuration); (2)

$C_{areal,GCD} = (i\Delta t)/(A\Delta V)$ (F cm^{-2}); (3)

$E = C_{areal,GCD}\Delta V^2/7.2$ (mWh cm^{-2}); (4)

$P = 3.6E/\Delta t$ (mW cm^{-3}). (5)

Where m is the solid content of MXene hydrogel electrode, I is current, s is the scan rate, ΔV is the potential window, A is the area of MSC electrodes, i is the applied current, and Δt is the discharge time.

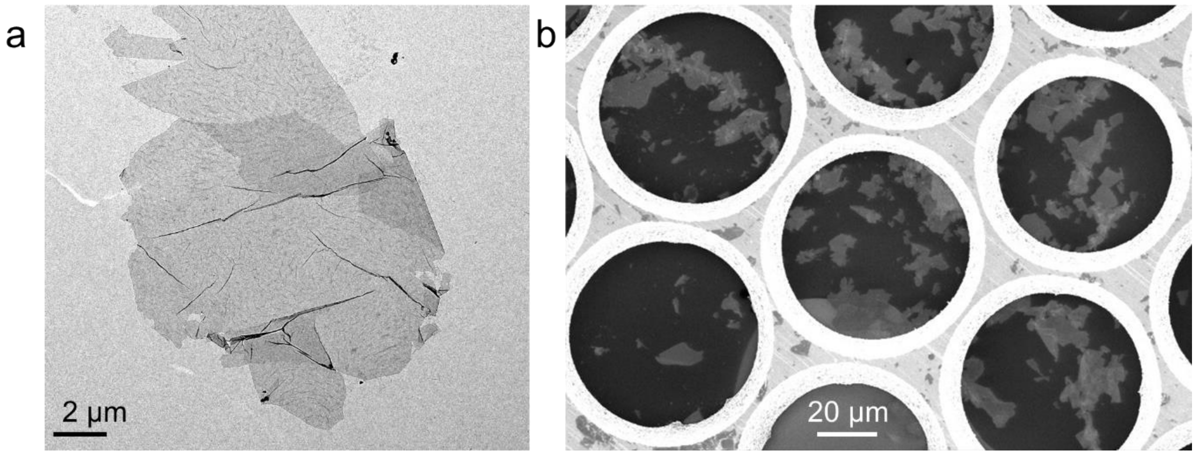

Figure S1. (a) The TEM and (b) SEM image of large MXene flakes for statistical purposes.

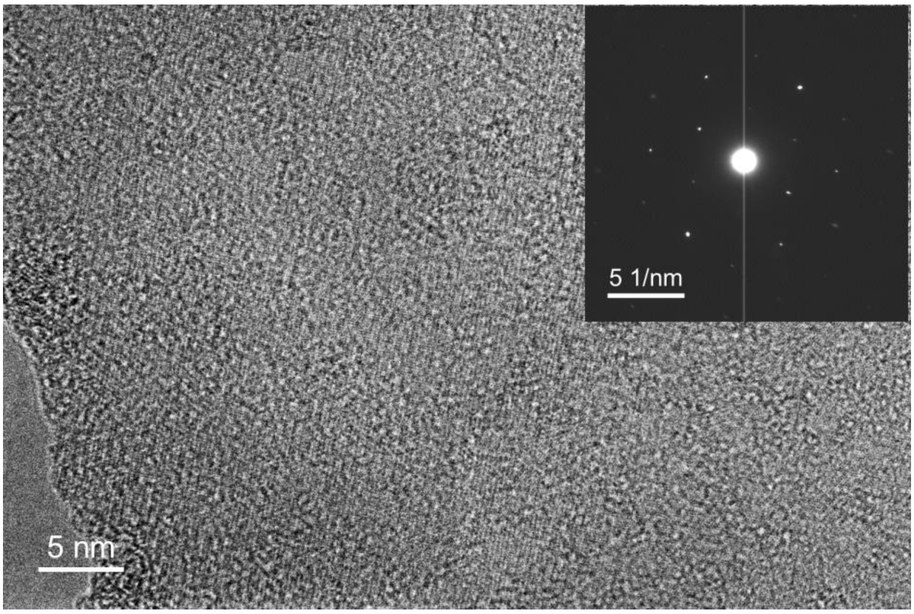

Figure S2. The HRTEM and (inset) SAED image of MXene flakes.

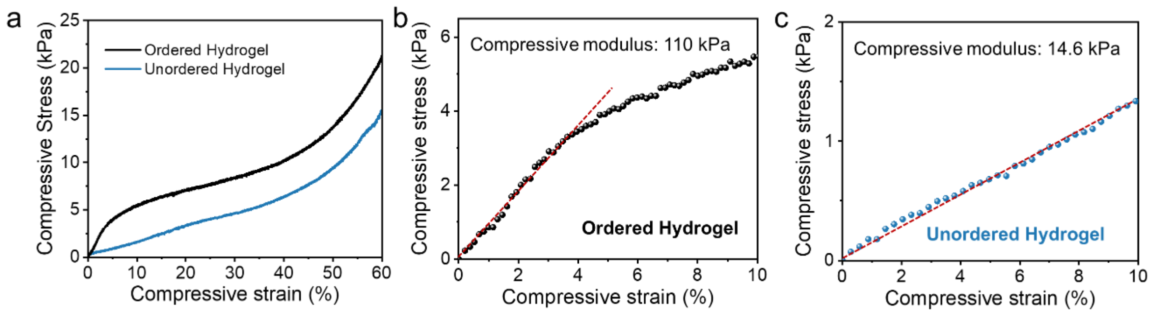

Figure S3. (a) The compressive stress-strain curves and compressive modulus of (b) ordered and (c) unordered MXene hydrogel.

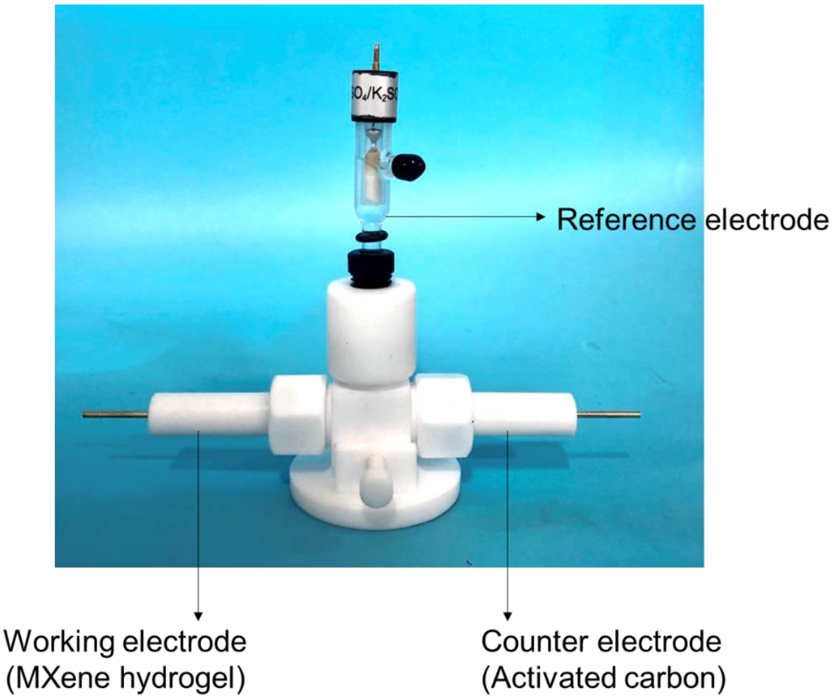

Figure S4. Swagelok-type cell with three-electrode setup.

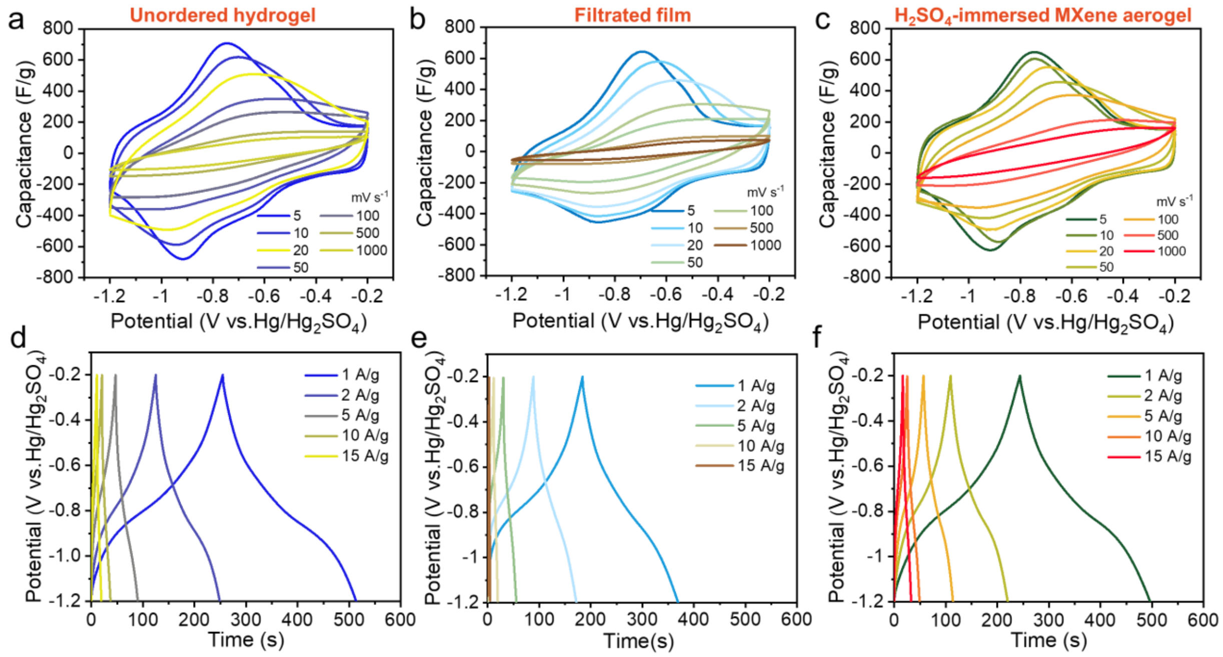

Figure S5. (a, b, and c) CV curves and (d, e, and f) GCD profiles of unordered hydorgel, filtrated film, and H_2SO_4-immersed MXene aerogel, respectively.

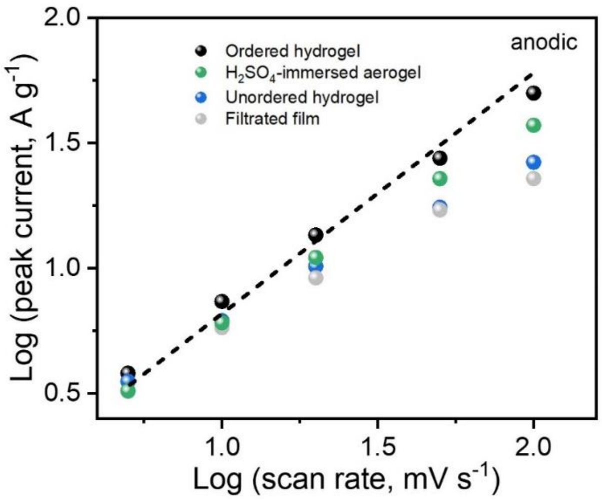

Figure S6. Plot of the anodic peak current against the scan rate for the four MXene samples.

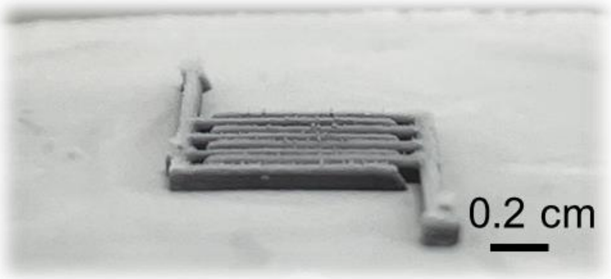

Figure S7. Unidirectional-freezing MXene MSC.

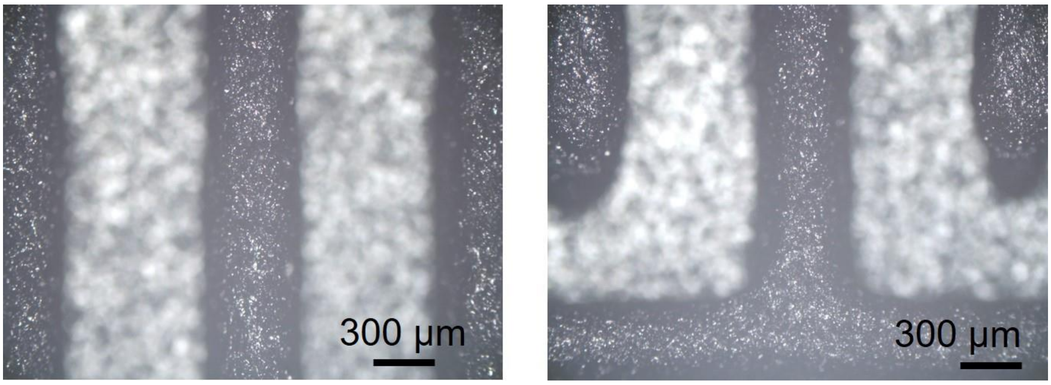

Figure S8. The optical microscope photographs of 3D-printed MXene MSC.

Table S1. The comparison of MXene-based MSCs electrochemical performance.

Materials	Methods	Electrolyte	Areal capacitance (mF cm^{-2})	Rate performance (mF cm^{-2})	Energy density (μWh cm^{-2})	Power density (mW cm^{-2})	Ref.
$Ti_3C_2T_x$	3D-printing	PVA/H_2SO_4	1035 (2 mV s^{-1})	4.68 (500 mV s^{-1})	51.7	5.7	[1]
$Ti_3C_2T_x$	3D-printing	PVA/H_2SO_4	2100 (1.7 mA cm-2)	1000 (42.7 mA cm^{-2})	24.4	0.64	[2]
MXene-AgNW-MnONW-C60	3D-printing	PVA/KOH	216.2 (10 mV s^{-1})	72.8 (2000 mV s^{-1})	19.2	58.3	[3]
MXene sediment	Screen-printing	PVA/H_2SO_4	158 (0.08 mA cm^{-2})	127 (2.4 mA cm^{-2})	1.64	0.778	[4]
$Ti_3C_2T_x$	Laser-scribing	PVA/H_2SO_4	25 (20 mV s^{-1})	10 (1000 mV s^{-1})	0.77	46.6	[5]
$Ti_3C_2T_x$	Stamping	PVA/H_2SO_4	61 (0.025 mA cm^{-2})	50 (0.8 mA cm^{-2})	0.63	0.33	[6]
$Ti_3C_2T_x$	Extrusion-printing	PVA/H_2SO_4	43 (0.005 mA cm^{-2})	33 (0.05 mA cm^{-2})	0.32	0.0114	[7]
$Ti_3C_2T_x$ /RuO_2	Laser-engraving	1 M H_2SO_4	60 (5 mV s^{-1})	50 (20 mV s^{-1})	19	1.5	[8]
$Ti_3C_2T_x$ /Be^{2+}	Template-depositing	$ZnSO_4$/Gelatine	77.2 (5 mV s^{-1})	31 (100 mV s^{-1})	3.86	0.12	[9]
$Ti_3C_2T_x$ /CNF	Template-depositing	PVA/H_2SO_4	25.3 (2 mV s^{-1})	13 (200 mV s^{-1})	0.08	0.145	[10]
$Ti_3C_2T_x$ /CNT	Extrusion-printing	PVA/H_3PO_4	30.8 (0.025 mA cm^{-2})	20 (0.4 mA cm^{-2})	8.37	17.3	[11]
**$Ti_3C_2T_x$**	**3D-printing and UFAT method**	**PVA/H_2SO_4**	**2004 (1.2 mA cm^{-2})**	**1202 (60 mA cm^{-2})**	**100**	**0.38**	**This work**

Movie S1. 3D printing all-MXene MSC.

References

[1] J. Orangi, F. Hamade, V. A. Davis, M. Beidaghi, *ACS Nano* **2020**, 14, 640.

[2] W. Yang, J. Yang, J. J. Byun, F. P. Moissinac, J. Xu, S. J. Haigh, M. Domingos, M. A. Bissett, R. A. W. Dryfe, S. Barg, *Adv. Mater.* **2019**, 31, 1902725.

[3] X. Li, H. Li, X. Fan, X. Shi, J. Liang, *Adv. Energy Mater.* **2020**, 10, 1903794.

[4] S. Abdolhosseinzadeh, R. Schneider, A. Verma, J. Heier, F. Nüesch, C. Zhang, *Adv. Mater.* **2020**, 32, 2000716.

[5] N. Kurra, B. Ahmed, Y. Gogotsi, H. N. Alshareef, *Adv. Energy Mater.* **2016**, 6, 1601372.

[6] C. Zhang, M. P. Kremer, A. Seral-Ascaso, S.-H. Park, N. McEvoy, B. Anasori, Y. Gogotsi, V. Nicolosi, *Adv. Funct. Mater.* **2018**, 28, 1705506.

[7] C. Zhang, L. McKeon, M. P. Kremer, S.-H. Park, O. Ronan, A. Seral‐ Ascaso, S. Barwich, C. Ó. Coileáin, N. McEvoy, H. C. Nerl, B. Anasori, J. N. Coleman, Y. Gogotsi, V. Nicolosi, *Nat. Commun.* **2019**, 10, 1795.

[8] Q. Jiang, N. Kurra, M. Alhabeb, Y. Gogotsi, H. N. Alshareef, *Adv. Energy Mater.* **2018**, 8, 1703043.

[9] S. Li, Q. Shi, Y. Li, J. Yang, T.-H. Chang, J. Jiang, P.-Y. Chen, *Adv. Funct. Mater.* **2020**, 30, 2003721.

[10] W. Tian, A. VahidMohammadi, M. S. Reid, Z. Wang, L. Ouyang, J. Erlandsson, T. Pettersson, L. Wågberg, M. Beidaghi, M. M. Hamedi, *Adv. Mater.* **2019**, 31, 1902977.

[11] J. Zhao, Y. Zhang, Y. Huang, X. Zhao, Y. Shi, J. Qu, C. Yang, J. Xie, J. Wang, L. Li, Q. Yan, S. Hou, C. Lu, X. Xu, Y. Yao, *J. Mater. Chem. A* **2019**, 7, 972.
